# Early health education and cognitive inhibitory control in children: a 20-year pilot study using the go/no-go task in Japan

**DOI:** 10.3389/fpubh.2025.1703017

**Published:** 2025-11-20

**Authors:** Noriaki Watanabe, Masayoshi Kamijo, Kazuki Ashida, Fumihito Sasamori, Masao Okuhara, Suchinda Jarupat Maruo, Hisaaki Tabuchi, Koji Terasawa

**Affiliations:** 1 Graduate School of Medicine, Science and Technology, Shinshu University, Ueda, Japan; 2 National Institute of Technology, Nagano College, Nagano, Japan; 3 Faculty of Engineering, Shinshu University, Nagano, Japan; 4 Department of Applied Information Engineering, Faculty of Engineering, Suwa University of Science, Chino, Japan; 5 Department of Occupational Health and Safety, Faculty of Public Health, Mahidol University, Bangkok, Thailand; 6 Department of Psychology, University of Innsbruck, Innsbruck, Austria; 7 Graduate School of Medicine, Shinshu University, Matsumoto, Japan

**Keywords:** early health education, cognitive inhibitory control, go/no-go task, health behavior, children

## Abstract

Extending healthy life expectancy requires attention not only to physical fitness but also to the development of cognitive self-regulation skills during childhood, which play a critical role in establishing lifelong health behaviors. Early health education has traditionally emphasized physical activity and lifestyle, yet cognitive aspects of self-control and inhibitory processes have received comparatively little attention. Inhibitory control is a central component of executive function, enabling children to regulate impulses, follow rules, and adapt to changing environments. Deficits in this capacity are associated with academic challenges, risk-taking behaviors, and poorer health outcomes later in life. Thus, incorporating assessments of cognitive inhibitory control into early health education may provide valuable insights for both educational and preventive health strategies. This pilot longitudinal study examined the feasibility of using the go/no-go task to assess inhibitory control in children aged 3–14 years in Nagano Prefecture, Japan. Assessments were conducted in 1998, 2008, and 2018, enabling exploration of changes over two decades. The task included three phases—formation, differentiation, and reverse differentiation—allowing for evaluation of both reaction times and error rates as indicators of speed–accuracy trade-offs in inhibitory performance. Results showed that children in 2018 exhibited significantly shorter reaction times but higher error rates compared with those assessed in 2008, suggesting a shift toward prioritizing speed over accuracy. These changes may reflect broader environmental and behavioral influences, such as increased exposure to digital devices, altered patterns of daily activity, or evolving educational contexts. Importantly, these findings indicate that inhibitory control, as captured by a simple cognitive paradigm, can reveal population-level shifts in child development over time. Incorporating cognitive tasks such as the go/no-go paradigm alongside conventional physical fitness testing in school-based health education may therefore enrich understanding of children's self-regulatory capacity. This approach has the potential to strengthen early identification of cognitive and behavioral trends, support tailored educational interventions, and inform broader community health promotion programs. By linking cognitive development with public health practice, it may contribute to strategies aimed at extending healthy life expectancy across the lifespan.

## Introduction

The WHO (1) reports that regular physical activity is effective in preventing and treating non-communicable diseases (NCDs), such as heart disease, stroke, diabetes, breast cancer, and colorectal cancer; preventing hypertension, overweight, and obesity; and improving mental health, quality of life (QOL), and well-being. However, the WHO (2) also suggests that 70% of the world's population does not achieve 150 min of moderate physical activity per week. Therefore, the WHO (3, 4) set a goal to reduce this number by 15% by 2030. However, it is estimated that it is difficult for people around the world to maintain exercise habits. Since 1988, five districts in Nagano Prefecture, Japan, have implemented comprehensive social capital-oriented health education based on incorporating dementia prevention activities into systematic health education programs for older adult individuals and promoting empathy and collaboration (5–9). Therefore, to examine the implementation of health education, we utilized the PDCA (Plan, Do, Check, Action) cycle, which is an incentive requirement of the international standard ISO9001, ranging from checking on planning health education to reviewing after implementation (we obtained ISO9001 certification in 2014 and moved to self-declaration in 2021) (10–16).

In 1940, the Ministry of Education and the Ministry of Health, Labour and Welfare (17, 18) conducted physical fitness tests for 17–19-year-olds to improve physical fitness and prevent tuberculosis. Afterward, the jurisdiction of physical fitness tests was transferred from the Ministry of Education to the Sports Agency (19); since 2008, these tests have been conducted for 6–79-year-olds with the aim of maintaining and promoting lifelong health from childhood to old age. Therefore, it is necessary to consider the health of older adult individuals, occupational health, and early childhood health. Among these aspects, research has shown that participation in comprehensive early-stage health education programs reduces the likelihood of indulging in unhealthy habits and excessive alcohol, tobacco, and illegal drug use (20). Hence, the effectiveness of early health education from an early age has been examined (21, 22). However, to date, no established health education methods have been reported for early childhood, school-age, or adolescent individuals. In addition, health education for older adult individuals, along with physical fitness testing, is useful as a screening test for dementia as a form of cognitive inhibitory control. However, go/no-go tasks such as cognitive inhibitory control have not been implemented alongside physical fitness tests for early childhood, school-age, and adolescent individuals. In this study, the foundation of health promotion that has been developed through school-based physical fitness testing in Japan since 2008 was regarded as the basis of early health education. The study explored the possibility of positioning this framework as an educational approach for fostering self-regulation and inhibitory control in children. The go/no-go task was introduced as an objective tool for evaluating these cognitive abilities, and the study was conducted as a pilot investigation to examine its feasibility. Because this task has been used in health programs for older adult populations, the present study aimed to confirm its applicability to early childhood and school-age populations. The go/no-go paradigm has been widely used to evaluate age-related differences in inhibitory control and executive functioning, as performance in this task reflects developmental changes in attention, self-regulation, and response inhibition across the lifespan (5–7, 9–16). By assessing accuracy and reaction time at different ages, this task can identify specific stages of cognitive development and detect shifts in the balance between speed and accuracy. These features make it a valuable tool for examining how cognitive inhibitory control evolves from early childhood to older adulthood. This addition clarifies the relevance of the go/no-go task for identifying developmental stages of inhibitory control and aligns the present study with its broader application across the lifespan. Accordingly, the term health education was retained in the title to reflect the broader educational and public health significance of this work.

Therefore, the purpose of this study is to examine whether go/no-go tasks can be feasibly used as a health education program to measure cognitive inhibitory control in early childhood, school-age, and adolescent individuals in addition to physical fitness tests, as seen in the older adult population.

## Methods

### Participants

This longitudinal study was conducted three times over a 20-year period—in 1998, 2008, and 2018—targeting children in Nagano Prefecture, Japan. A go/no-go task was employed as a measure of cognitive inhibitory control in a pilot study aimed at informing future developments in early health education. The study included children attending the same affiliated preschools, elementary schools, and junior high schools in Nagano Prefecture.

In 1998, children in the first grade of elementary school were aged 6–7 years, born between April 2, 1992, and April 1, 1993; for consistency, their age was recorded as 6 years. The age for second-grade elementary school students was similarly set at 7 years.

Participation in parent meetings was voluntary, and non-participation had no negative consequences. Parents received detailed explanations about the study objectives and procedures, including potential risks, prior to participation. All methods were conducted in accordance with relevant guidelines and regulations. Written informed consent was obtained from all participants and their parents. Consent could be withdrawn at any time without penalty. Parents were assured that all data would be kept strictly confidential and handled securely.

The study protocol was approved by the Ethics Committee of Shinshu University (approval numbers: 003 and 180; approval dates: July 14, 2007, and April 14, 2012, respectively).

### Brain function tests

This method was originally developed by Masaki and Moriyama in 1989 (23) and later applied by Terasawa et al. in 2014 (24), confirming its feasibility among young participants, and has since been adapted in subsequent studies (9, 24). Based on these previous studies, the present experiment followed the same basic procedure, which consisted of three components: formation, differentiation, and reverse differentiation.

In the formation experiment, participants were instructed to squeeze a rubber ball in response to randomly appearing red lights. Five trials were conducted.

In the differentiation experiment, participants squeezed the ball in response to a red light and refrained from squeezing in response to a yellow light. Twenty trials were conducted (10 per condition).

In the reverse differentiation experiment, participants were instructed to squeeze the ball in response to a yellow light and refrain from squeezing in response to a red light, with 20 trials conducted similarly. The stimulus durations ranged from 200 to 1,100 ms, and the interstimulus intervals varied randomly from 1,300 to 7,500 ms.

A miss was defined as a failure to squeeze the ball when required (go errors), and a mistake was defined as an incorrect squeeze when it was not required (no-go errors). We calculated the average reaction time for each of the three tasks, as well as the overall average reaction time. In addition, the total number of misses for the go tasks and the number of mistakes for the no-go tasks were recorded in both the differentiation and reverse differentiation conditions.

The go/no-go task was administered individually in a quiet classroom or gymnasium within each participating kindergarten or school. Participants were seated approximately 50 cm from the display monitor and instructed to press a button as quickly and accurately as possible in response to the stimuli presented on the screen. The stimuli were presented using the standardized Go/No-Go Task System Version 5.1, Faculty of Engineering, Shinshu University, Japan, which has been validated in previous studies involving both children and older adults (9–16). All sessions were conducted under standardized instructions and supervision by trained staff, following the same protocol during each measurement period 1998, 2008, and 2018.

Participants in each survey year were independently recruited, and no individuals were measured repeatedly across cohorts. Therefore, the data were treated as cross-sectional rather than longitudinal. One-way and two-way analyses of variance were used to examine differences by age group and measurement year. In future longitudinal studies, linear mixed-effects models will be considered to account for within-subject variability over time.

### Statistical analysis

This study included a total of 1,164 children aged 3 to 14 years, with 443 in 1998, 391 in 2008, and 330 in 2018. Because this was an exploratory longitudinal study aiming to assess feasibility rather than to test a specific hypothesis, no *a priori* power calculation was conducted. However, the total sample size exceeded 1,000 participants across the three survey periods, which is sufficient to detect medium effect sizes (η^2^ > 0.02) in repeated-measures ANOVA. These results can therefore serve as a reference for determining sample sizes in future confirmatory studies. Reaction times and related variables from the go/no-go tasks were analyzed using two-way repeated measures ANOVA, with survey year (1998, 2008, 2018) and participant age (3–14 years) as factors. When significant interactions were observed, simple main effects were examined. The Bonferroni method was used for multiple comparisons if significant effects were found. Reaction time data for 1998 were not available at all ages; thus, they were excluded from analysis.

All statistical analyses were conducted using SPSS Statistics version 29 (IBM Corp.). Statistical significance was set at *p* < 0.05.

## Results

**Figure 1:**
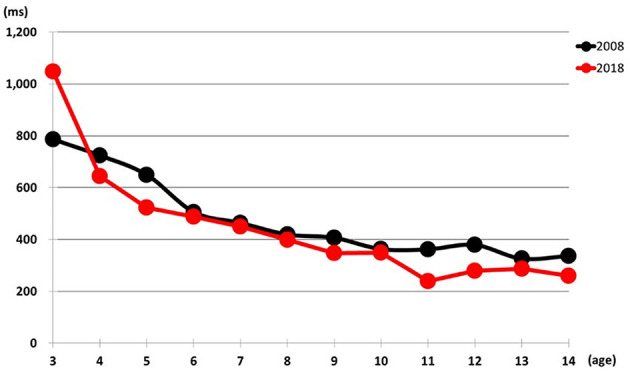
Comparison of average reaction times between 2008 and 2018.

The number of participants was 443 in 1998, 391 in 2008, and 330 in 2018, totaling 1,164 children aged 3 to 14 years (Table 1). Figure 1 illustrates the average reaction times for the formation, differentiation, and reverse differentiation tasks in 2008 and 2018.

**Table 1:** The number of participants by age in 1998, 2008 and 2018.

**Year/Age**	**3**	**4**	**5**	**6**	**7**	**8**	**9**	**10**	**11**	**12**	**13**	**14**	**Total**
1998	22	52	53	37	38	33	36	34	33	37	32	36	443
2008	24	39	29	32	35	28	28	34	30	38	38	36	391
2018	24	24	29	35	25	25	25	20	20	40	34	29	330

Values indicate the number of participants in each age group for the 1998, 2008, and 2018 surveys. Participants were independently recruited in each year, and no individuals were measured repeatedly across cohorts.

A significant interaction was observed in total reaction time [*F* (11,697) = 8.973, *p* < 0.001]. Specifically, 3-year-olds showed significantly longer reaction times in 2018 than in 2008 (1,048 ms vs. 786 ms, *p* < 0.001). In contrast, children aged 4, 5, 11, 12, and 14 years demonstrated significantly shorter reaction times in 2018 than in 2008 (*p* < 0.01 to *p* < 0.001), indicating a trend toward faster responses among older children in more recent years (Figure 1, Table 2).

**Table 2:** Relationship between in 2008 and 2018.

**Measurements**	**Source**	**df**	***F***	***p***	***ηp*2**	**Multiple comparison**
Total average reaction time	Year	1	13.867	0.000	0.020	3:2008 <2018, 4–5, 11–12, 14:2008 > 2018
	Age	11	131.149	0.000	0.674	2008:3 > 5–14, 4–5 > 6–14, 6 > 10–14, 7 > 10–11, 13–14
	Year × Age	11	8.973	0.000	0.124	2018:3 > 1 > 5–14, 5 > 8–14, 6 > 9–14, 7–8 > 11–14

Values are presented as mean (standard deviation). Reaction time is measured in milliseconds. Misses indicate go errors, and mistakes indicate no-go errors.

**Figure 2:**
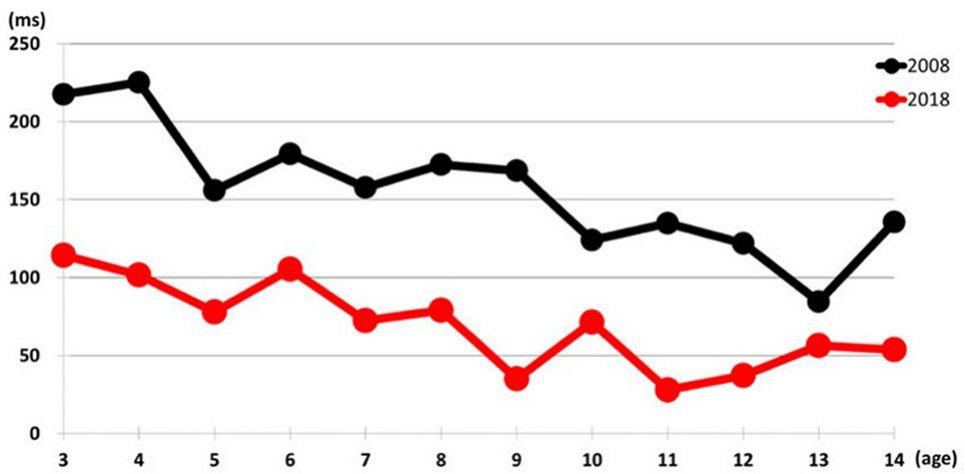
Comparison of differentiation minus formation reaction time in 2008 and 2018.

Regarding the difference in reaction time between differentiation and formation tasks, no significant interaction was observed [*F* (11,697) = 0.696, *p* = 0.743). However, the main effects revealed that the average difference was significantly smaller in 2018 (69 ms) compared to 2008 (156 ms), suggesting improved cognitive efficiency in more recent cohorts (Figure 2, Table 3).

**Table 3:** Relationship between in 2008 and 2018.

**Measurements**	**Source**	**df**	***F***	***p***	***ηp*2**	**Multiple comparison**
The difference time (differentiation – formation)	Year	1	72.969	0.000	0.095	2008 > 2018
	Age	11	3.309	0.000	0.050	3 > 12–13, 4 > 11–14
	Year × Age	11	0.696	0.743	0.011	

Values are presented as mean (standard deviation). Reaction time is measured in milliseconds. Misses indicate go errors, and mistakes indicate no-go errors.

**Figure 3:**
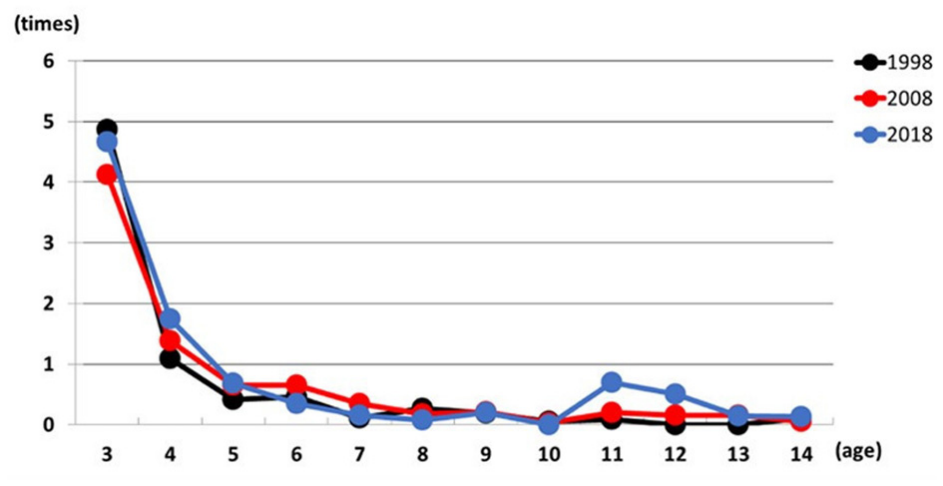
Relationship between total number misses and age in 1998, 2008, and 2018.

As shown in Figure 3, the total number of misses in differentiation and reverse differentiation tasks decreased with age across all 3 years. No significant interaction was found (*F* (11,697) = 0.651, *p* = 0.889], and children aged four and older consistently made fewer than two misses, indicating increased attentional control with age (Table 4).

**Table 4:** Results of two-way ANOVA for reaction time, misses, and mistakes by survey year and age group.

**Measurements**	**Source**	**df**	***F***	***p***	***ηp* ^2^**	**Multiple comparison**
Total number of misses	Year	2	1.080	0.340	0.002	
	Age	11	68.083	0.000	0.399	3 > 4 > 5–14
	Year × Age	22	0.651	0.889	0.013	

Values indicate *F* statistics from two-way analysis of variance (ANOVA) with survey year (1998, 2008, 2018) and age group (3–14 years) as factors. *p* < 0.05 was considered statistically significant.

**Figure 4:**
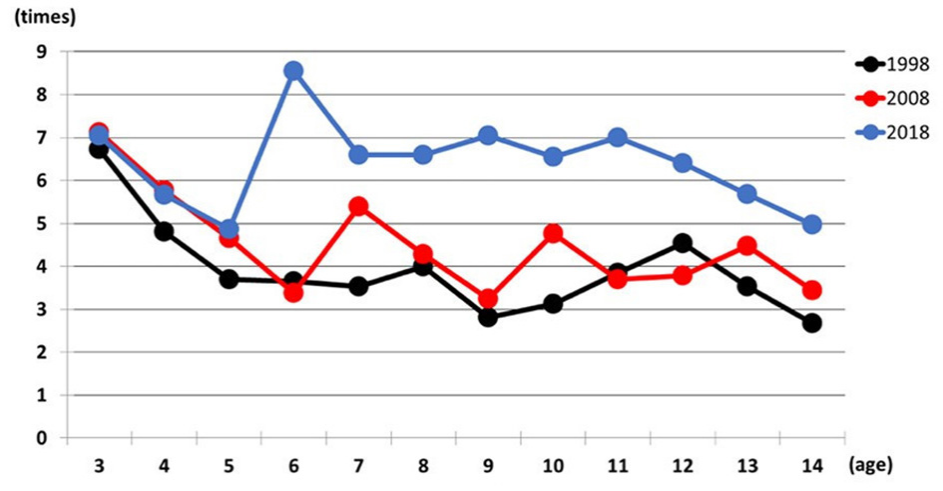
Relationship between total number mistakes and age in 1998, 2008, and 2018.

Figure 4 presents the total number of mistakes in differentiation and reverse differentiation tasks. A significant interaction was found [*F* (22,1128) = 2.074, *p* < 0.01]. Children aged 6, 8, 9, and 11 years showed significantly higher mistake rates in 2018 than in previous years. For example, 6-year-olds made 8.5 mistakes in 2018, compared to 3.6 in 1998 and 3.4 in 2008 (*p* < 0.001). Similar trends were seen in other age groups, including notable increases among 7, 10, and 13–14-year-olds in 2018 vs. 1998, and among 12-year-olds in 2018 vs. 2008. Additionally, 3-year-olds consistently had higher mistake counts than most other age groups in both 1998 and 2008, while 6-year-olds in 2018 outperformed 5, 13, and 14-year-olds (*p* < 0.05). These results indicate age-specific patterns in error rates that have shifted over time (Table 5).

**Table 5:** Relationship between in 1998, 2008 and 2018.

**Measurements**	**Source**	**df**	***F***	***p***	***ηp*2**	**Multiple comparison**
Total number of mistakes	Year	2	48.696	0.000	0.079	6, 8–9, 11:1998, 2008 <2018, 7, 10, 13–14:1998 <2018
	Age	11	4.092	0.000	0.038	12:2008 <2018 1998:3 > 5, 7, 9–10, 14
	Year × Age	22	2.074	0.003	0.039	2008:3 > 6, 9, 11–12, 14, 2018:6 > 5, 13–14

Values indicate F statistics from two-way analysis of variance (ANOVA) with survey year (1998, 2008, 2018) and age group (3–14 years) as factors. *p* < 0.05 was considered statistically significant.

## Discussion

This study was a pilot investigation aimed at supporting the future development of early health education. It was conducted longitudinally over a 20-year period (1998, 2008, and 2018) and involved children in Nagano Prefecture, Japan. The purpose was to assess whether health education incorporating cognitive inhibitory control tasks—similar to those used for the older adult—could be applied to younger populations using the go/no-go paradigm.

Our findings revealed significantly shorter reaction times in the differentiation-minus-formation task and a significantly higher number of mistakes in 2018 compared to 2008. This suggests that greater emphasis may have been placed on speed, potentially at the cost of accuracy. Prior studies indicate that inhibition is more difficult at faster response speeds (25, 26). The observed reductions in both differentiation and formation task times further suggest that the time required to process and respond to stimuli was shortened in 2018. Consequently, some decisions may have been made too hastily, leading to incorrect responses.

Environmental changes in children's lifestyles may partially account for these shifts. For example, brain function differs depending on whether one solves arithmetic problems mentally or with a calculator, with significantly lower cerebral blood flow observed during calculator use (27). Increasing activities that stimulate the brain may lead to more complex neural connections and enhanced cognitive function (28, 29).

Dual-task training in older adult adults, combining physical activity with mental tasks such as arithmetic, has demonstrated positive effects on go/no-go performance. One study reported that such training increased cerebral blood flow during the go task and reduced it during the no-go task (30). Initially, older adults showed faster reaction times but made more errors. After walking approximately 7,000 steps daily for a year, their error rates decreased even as decision-making time increased. Continued training in the second year further reduced both reaction times and errors due to improved decision accuracy (7).

These findings suggest that similar dual-task interventions—combining physical exercise and cognitive tasks—might be effective for children as well. While causality remains unclear, our results support the idea that early health education incorporating both physical and cognitive elements, such as the go/no-go task, may be valuable. These tasks could serve as effective tools for promoting self-regulatory development in children, analogous to their use in older adult health programs.

This pilot study did not aim to use the go/no-go task as a direct intervention tool, but rather to examine its feasibility as a cognitive assessment component within health education. Performance on the go/no-go task generally improves with age and develops in association with the maturation of neural circuits in the prefrontal cortex responsible for executive functions. These characteristics are consistent with previous studies showing that sustained attention and response inhibition gradually improve throughout development (31–33). Accordingly, this study positioned the go/no-go task as a potential framework for evaluating and understanding children's self-regulation and attention in early health education. The go/no-go task has long been applied in health education and cognitive assessments among older adults. Extending this framework to children and adolescents, the present study explored the possibility of incorporating cognitive components into early health education. Future research should develop specific training protocols that include diverse stimuli, increased trial numbers, and multiple sessions to examine the potential of the go/no-go task as a tool for improving inhibitory control. As information on participants' educational background and physical or mental health was not collected in this study, this has been identified as a limitation. These factors will be examined in future longitudinal studies to deepen the understanding of individual differences in cognitive development.

## Data Availability

The data analyzed in this study is subject to the following licenses/restrictions: The datasets generated and analyzed for this study are not publicly available due to ethical and privacy restrictions. Individual-level data contain sensitive information on children and cannot be shared openly. Requests to access the datasets should be directed to the corresponding author (kterasa@shinshu-u.ac.jp) and will be considered in accordance with institutional and ethical guidelines. Requests to access these datasets should be directed to kterasa@shinshu-u.ac.jp.
